# Curriculum reform: Why? What? How? and how will we know it works?

**DOI:** 10.1186/s13584-018-0221-4

**Published:** 2018-06-07

**Authors:** Shmuel Reis

**Affiliations:** Center for Medical Education, Hadassah/Hebrew University Faculty of Medicine, P.O.B 12272, 9112102 Jerusalem, Israel

**Keywords:** Medical education, Basic medical education, Curricular reform, Competency based medical education

## Abstract

In a recent IJHPR article, Dankner et al. describe a reform in one longitudinal strand within Basic Medical Education i.e.“ public health and preventive medicine curriculum” using a Competency Based Medical Education approach. This reform raises several concerns: What should prompt a medical school to change a curriculum? How should such change be conducted? What kinds of paradigms may inform such a change? What constitutes a success in a curricular reform? And, how can curricular reform be evaluated within a reasonable time framework?

This commentary addresses these concerns and concludes that curricular reform should follow as much as possible the current wisdom of educational innovation and change strategy, follow a clear vision, mission, and selected educational paradigm, and pay attention to stakeholders, context, culture and politics. The design should allow for the emergence of unintended consequences. Implementation needs careful planning and monitoring and the evaluation should be multi-faceted. Finally, since all Israeli medical schools are now using the Competency Based Medical Education approach and aligning their curricula and testing accordingly, a fascinating collaborative opportunity exists to professionalize this process and hopefully make a positive impact.

## Background

Curricular reform in Basic Medical Education (BME i.e. medical school) has been a hot topic for quite some time. New medical schools strive to embody innovation, and seasoned ones are seeking compliance with current principles of adult learning, the needs of society, the changing nature of healthcare and how current students learn best. Israel is no exception, with a new and innovative medical school now into its sixth year (Safed) and all four of the others having already gone through at least one curricular overhaul in the last decade. In a recent IJHPR article, Dankner et al. describe a reform in one longitudinal strand within BME i.e.“ public health and preventive medicine curriculum, during 2013- 2014, according to the competency-based medical education (CBME) ... aimed to strengthen competencies… (of) epidemiology and statistics for appraisal of the literature and implementation of research; the application of health promotion principles and health education strategies in disease prevention; the use of an evidence-based approach in clinical and public health decision making; the examination and analysis of disease trends at the population level; and knowledge of the structure of health systems and the role of the physician in these systems” [1].

This reform raises several concerns: What should prompt a medical school to change a curriculum (whether a reform of the full curriculum or one limited to a specific course)? How should such change be conducted? What kinds of paradigms may inform such a change? What constitutes a success in a curricular reform? And, how can curricular reform be evaluated within a reasonable time framework? This commentary addresses all/several of these concerns.

## What should prompt a medical school to change a curriculum (whether a reform of the full curriculum or one limited to a specific course)?

The literature is replete with reasons for radical change in the traditional medical school curriculum [2, 3] that was launched over a 100 years ago through the Flexner report. A hundred years later, both healthcare and learning have been transformed [3]. The focus of care has transitioned from acute to chronic conditions, from hospital to the community, with technology transforming care and learning. Student centeredness (analogous to patient centeredness) has replaced teacher centeredness, eLearning is replacing lecture based teaching, and new paradigms such as competency/outcome based education are replacing content or time based education [2–4]. Curricula are called upon to comprise standardized outcomes, yet allow individualized learning trajectories; support self- regulated learning and foster curiosity; promote professional identity formation as their major goal, and contextualize learning through early clinical exposure, longitudinal experiences and service learning [4].

Dankner et al. aimed to “*evaluate and update objectives* for the public health and epidemiology curriculum for medical students; to *review and revise* the current curriculum; to *introduce a revised curriculum* in public health; and to *introduce appropriate teaching methods in accordance with the competency-based medical education* (CBME) approach” within a larger curricular reform [1]. The authors present a detailed and appropriate rationale that includes both the transformed content of preventive medicine, epidemiology and public health as well as a transformation of medical education at its core. This approach is supported by Borkan et al. [5] when advocating for a circumscribed, rather than an entire medical school curriculum reform, based on their experience in introducing a Health Systems Science innovative program for a cohort within their medical school.

## How should such change be conducted?

Kern’s six steps are often used as a guide for curricular design. These are: 1) Problem identification and general needs assessment 2) Needs assessment for targeted learners 3) Goals and objectives 4) Educational strategies 5) Implementation 6) Evaluation and feedback [6]. However, curricular reform is not just about the technical pedagogical aspect. Change, especially in a complex system such as a medical school, is fraught with resistance, inertia, power and ego struggles which call for a strategic approach as well. Appointing a dedicated committee, submitting a proposal and receiving an approval from the governing parties is just the tip of the iceberg. McKimm & Jones [7] offer 12 tips that expand on Kern’s roadmap and which shed light on the hidden part of the iceberg. Their tips include: Create the vision, aligned to mission; Develop a strategy for change involving key stakeholders; Quick visible wins and communication are vital; Analyze the internal environment and culture; Consider the external environment, cultural contexts, and political influences. These aspects: vision and mission, change strategy, accounting for the different stakeholders, quick wins, considerations of context, culture and especially local and outside politics, and more are the hallmarks of an informed approach to curricular change. Recently, Velthuis Floor et al. conducted an in-depth inquiry into a curricular reform identifying 3 major challenges: the large number of stakeholders championing a multitude of perspectives, dealing with resistance, and steering the change process [8].

Thus, the medical education literature fortunately supplies reformers with reasonable, practical guidelines as well as exemplars, spanning both aspects (design and change strategies) of such endeavors [5–8].

## What kinds of paradigms may inform such a change?

The authors use the competency/outcome based medical education (CBME) paradigm which indeed is currently the most visible paradigm [2, 3, 9]. It emerged from “ (i) the redefinition of the doctor, which includes features previously not emphasized, and (ii) the strong wish to certify doctors based on outcomes (i.e. attained competence), rather than inputs (i.e. time in training, rotations completed, etc.)” [10]. In the last 40 years, several paradigm shifts were postulated in medical education i.e. the bio-psychosocial model, patient and student centeredness, problem based and system based curricula. In the March 2108 issue of Academic Medicine, an additional elaboration on CBME (time variable CBME) is presented. The challenge presented by this new paradigm is not merely a technical one, as it entails profound transformations, requires new competencies, creates disequilibrium, resistance, a sense of loss and often takes longer than technical change [8]. Also, although we are fortunate to be guided for these challenge by existing literature, critics still question the paradigm’s rationale and solid evidence that it makes a positive difference is still lacking [11–13].

## What constitutes a success in a curricular reform?

A curricular reform that is CBME-informed should be easy to evaluate. When outcomes and competencies are described behaviorally, their assessment flows directly. Currently, CBME programs have included entrustable professional activities (EPAs) as milestones in the progression towards mastering a competency [14, 15]. In addition to assessing individual students’ learning, an evaluation of the new curriculum is warranted. In the article by Dankner et al. [1], the proposed evaluation consists of student satisfaction end of course surveys, a comparison of knowledge levels between the graduates of the old and new curricula and an evaluation of MD thesis quality before and after the intervention. These are necessary components that go beyond the usual “happiness” index of students’ surveys. Nevertheless, current recommendations of curricular reform suggest that it is important to also pay attention to process (i.e. effectiveness, implementation process and fit with goals) outcome (effects on participants’ learning, categorized as instructional or nurturant) and impact (longer term program effects) [16].

Moreover, curricular innovation should call forth evaluation innovation, which may entail attention also to outcomes such as professional identity formation, professionalism and commitment to social accountability. Formative assessment may be harnessed to both enhance learning (when applied through the learning process) and make the hidden curriculum explicit [17, 18]. In the present case, the longitudinal, 6 year-long curriculum lends a special opportunity to monitor the learning and program developmentally, i.e. measure increments in knowledge, attitudes and skills over time and evaluate the graduate’s competencies at graduation and possibly in subsequent stages of the professional life cycle.

## How can it curricular reform be evaluated within a reasonable time framework?

Educational interventions are notoriously difficult to evaluate [16]. It takes at least a decade to design and implement an entire medical school curriculum. Evaluation needs to be planned and implemented for longer than this time framework, a rare and unusual event. The same will be true for a longitudinal strand such as the public health/ health promotion curriculum described by Dankner et al. [1]. Monitoring the program and the learners for the duration of 6 years, comparing to the former curriculum and looking for transfer to the work-place as well as impact on practice and care delivery requires a robust infrastructure and a long haul approach (10–20 years, [16–19]). Nevertheless, reports of shorter term evaluations exist; they employ methods such as portfolios, evaluation of faculty development and teachers’ perceptions in the new curriculum, interviews of teachers, learners and education managers, longitudinal participant observation in teaching, and measuring educational climate [17–19].

## Conclusions

Curricular reform, be it of an entire medical school curriculum or a significant longitudinal component should follow as much as possible the current wisdom of educational innovation and change strategy. It should follow a clear vision and mission, a selected educational paradigm, and pay attention to stakeholders, context, culture and politics. It goes beyond the technical and is complex. As such, a buy-in, strong leadership support and early wins are paramount. The design should allow for the emergence of unintended consequences. Implementation needs careful planning and monitoring and the evaluation should be multi-faceted, employing a mixed-method innovative design with short- and long-term components. Since all Israeli medical schools are now using the CBME approach and aligning their curricula and testing accordingly, a fascinating collaborative opportunity exists to professionalize this process and hopefully make a positive impact.

## Abbreviations

BME: Basic medical education (medical school)
CBME: Competency based medical education
EPAs: Entrustable professional activities
IJHPR: Israel journal of healthcare policy research
MD: Medical doctor
